# Exploring Individual Factors Affecting Endothelial Function Response Variability in Aging: Implications for Precision Nutrition

**DOI:** 10.3390/nu17142285

**Published:** 2025-07-10

**Authors:** Emily K. Woolf, Leanne M. Redman

**Affiliations:** Reproductive Endocrinology and Women’s Health Laboratory, Pennington Biomedical Research Center, Baton Rouge, LA 70808, USA; emily.woolf@pbrc.edu

**Keywords:** endothelial function, healthy aging, precision nutrition, nutrition and aging, interindividual variability, flow-mediated dilation

## Abstract

Aging is a major non-modifiable risk factor for cardiovascular disease (CVD), in part due to its detrimental effects on vascular endothelial function. Dietary interventions, including those rich in plant-based components or following dietary patterns such as the Mediterranean Diet, have been shown to improve endothelial function in older adults, assessed via brachial artery flow-mediated dilation (FMD). However, it is well recognized that FMD responses to dietary interventions often show considerable variability among individuals. This variability presents a major challenge to translating findings into effective, population-level guidance highlighting the need for more tailored approaches for CVD risk prevention. Thus, to advance these precision nutrition approaches, research must move beyond the overall group mean effects and begin to investigate the factors driving this variability. This narrative review summarizes current evidence on nutritional interventions that improve endothelial function with aging, highlights potential contributors to individual response variability, and outlines future research directions to reduce this variability to enhance clinical relevance and advance precision nutrition for the aging population.

## 1. Introduction

**Figure 1:**
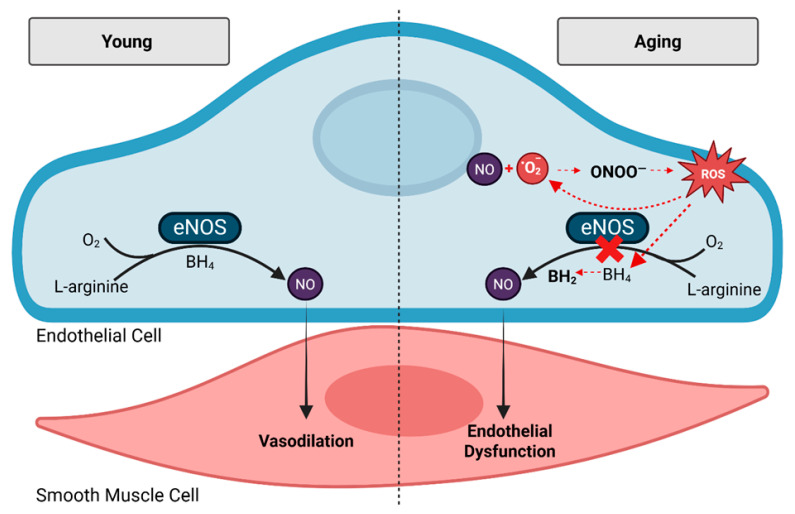
Mechanisms for NO-mediated endothelial function under young and healthy conditions and oxidative stress-mediated endothelial dysfunction as seen in the aging population. Abbreviations: endothelial nitric oxide, eNOS; nitric oxide, NO; oxygen, O_2_; peroxynitrite, ONOO^−^; reactive oxygen species, ROS; superoxide, O_2_^−^; tetrahydrobipterin, BH_4_.

Cardiovascular disease (CVD) is the leading cause of death in the United States, affecting nearly half of all adults [1]. Aging is the most significant non-modifiable risk factor for CVD. With over 80 million adults projected to be ≥65 years old by 2050, the burden of CVD is expected to continue to rise [1,2,3,4,5]. A hallmark of CVD development is vascular endothelial dysfunction, characterized by impaired vasodilation and the reduced bioavailability of the critical endothelium-derived vasodilator, nitric oxide (NO) [6]. Endothelial dysfunction not only precedes CVD but also predicts its progression [7]. Under healthy conditions, endothelial nitric oxide synthase (eNOS) produces NO, which diffuses into vascular smooth muscle cells to promote vasodilation and maintain blood flow (Figure 1) [8]. NO also exerts anti-inflammatory and antithrombic effects, making it not only a critical molecule for endothelial function, but also for overall cardiovascular health [9].

Unfortunately, vascular endothelial dysfunction occurs naturally with aging. Advancing age is associated with decreased NO production and bioavailability (Figure 1), largely due to increased oxidative stress which is a well described hallmark of natural mammalian aging [4,10,11,12,13]. Excess reactive oxygen species uncouple eNOS, leading to superoxide rather than NO production [6,14]. Superoxide reacts with NO to form peroxynitrite, a damaging molecule that further reduces NO availability and worsens endothelial function (Figure 1) [6,14]. Thus, strategies that mitigate oxidative stress and preserve NO are crucial during aging.

Endothelial function is commonly assessed in clinical research settings using brachial artery flow-mediated dilation (FMD), which measures the percent change in artery diameter in response to increased blood flow [15]. Although no standardized FMD cutoffs exist, higher FMD values generally indicate better endothelial function. Notably, each 1% increase in FMD is associated with a 7% reduction in relative risk for CVD [1,15]. Research shows that aging-induced FMD impairments are largely driven by increased oxidative stress and reduced NO bioavailability [16]. Hence, older adults exhibit significantly lower FMD values compared to their younger counterparts, even in the absence of comorbidities and disease [4]. Importantly, endothelial dysfunction assessed via FMD has been implicated not only in CVD progression but also in the progression of other age-related conditions such as cognitive decline and chronic kidney disease [17,18], highlighting its systemic relevance in aging populations. This emphasizes the need for early and targeted interventions to maintain FMD among aging adults.

Diets that are rich in plant-based foods (e.g., fruits, vegetables, nuts) contain bioactive compounds such as (poly)phenols, L-arginine, and nitrates which play an important role in reducing oxidative stress and supporting NO production and bioavailability [19,20,21,22,23]. However, variability in individual responses remains an important limitation in all clinical research, suggesting that a one-size-fits-all dietary recommendation is suboptimal for aging individuals. This individual variability may not stem solely from extrinsic differences (e.g., lifestyle) but may also be influenced by identifiable and measurable intrinsic factors as well (e.g., metabolism, menopause status). Thus, this narrative review explores potential contributors to variability in FMD responses to dietary interventions in aging adults, with the goal of informing future precision nutrition research strategies to optimize endothelial function during aging.

## 2. Narrative Review Rationale and Literature Search

This review explores possible biological, physiological, and lifestyle factors that may influence FMD responses to dietary interventions, offering insights that can inform the design and analysis of future precision nutrition research.

To lay the foundation that dietary interventions improve FMD in aging populations, first this review identifies the human randomized controlled trials (RCTs) in which dietary interventions led to an overall statistically significant improvement in FMD in aging adults. For this, only RCTs that had an aging study population inclusion criteria of ≥45 years old were reported, as this period encompasses accelerated physiological aging processes (e.g., oxidative stress, inflammation, hormonal shifts such as menopause) that are known to impact endothelial function and FMD [4,6,16,24,25,26]. While the choice of 45 years as a lower age limit is somewhat arbitrary and varies across studies, it reflects a balance between biological relevance and the availability of research populations meeting this criterion. Second, our review explores whether these studies reported individual variation in response to the intervention. Individual variability was identified based on the explicit reporting of variation in the original studies or was inferred from visual or descriptive data (e.g., a large spread in outcome measures). No additional statistical analyses were performed.

This review focuses on FMD as the only measure of endothelial function due to its status as the non-invasive gold standard in clinical research, with well-established associations to CVD risk and prevention [27,28,29,30]. Alternative endothelial function assessments (e.g., peripheral arterial tonometry; EndoPAT) were excluded to maintain consistency, as they assess different aspects of endothelial function (e.g., macro- vs. microvascular function) [31]. Studies that failed to demonstrate a significant group-mean improvement in FMD following a dietary intervention were not included. Studies using foods, beverages, food-based products (e.g., nutritional gels), and dietary patterns were included for their applicability to real-world dietary strategies and public health applications. Additionally, a search was carried out to examine studies that highlight potential factors that could contribute to FMD response variability to dietary interventions in aging populations (e.g., microbiome, pre-existing comorbidities, metabolomics, dietary adherence). As research is very limited, for this final component, the literature search included any form of dietary intervention (e.g., whole foods, extracts, supplements). Thus, a literature search was conducted using Google Scholar and PubMed to identify studies that fit these criteria related to dietary interventions and FMD outcomes in aging populations. While clinical implementation of these factors is outside the scope of this review, the identification of key response-modifying factors represents a foundational step toward developing precision nutrition strategies.

As a narrative review, this work is subject to limitations including potential publication bias, the incomplete retrieval of all relevant studies, and the subjective selection of the included research. These factors could influence the comprehensiveness and generalizability of the findings. Nonetheless, the narrative approach allows for a focused exploration of key factors that could be influencing variability in FMD responses to dietary interventions in aging adults, laying important groundwork for future research focusing on advancing the field of precision nutrition.

## 3. Foods and Dietary Patterns That Improve Flow-Mediated Dilation in Aging Adults

Several foods and dietary patterns have been identified to improve FMD in aging adults (Table 1). In three different RCTs, blueberry consumption for three to six months improved FMD from 0.86% to 1.45% units in older adults with increased CVD risk [14,23,32,33]. However, Woolf et al. (2023) reported large variability in the change in FMD with blueberry consumption between individuals, with change values ranging from −4.75% to +8.0% units pre- to post-intervention [23]. Blueberries contain (poly)phenols such as anthocyanins, which have been shown to improve FMD by increasing NO production through reducing oxidative stress, upregulating eNOS [14], and/or by binding to estrogen receptors on endothelial cells to activate eNOS [34,35]. Additionally, an RCT showed that daily consumption of 67 g of soy nuts for 16 weeks improved FMD by 1.48% units in healthy older adults. However, responses varied widely, with individual changes in FMD ranging from 0% to 15% pre- to post-intervention [36]. Soy nuts are high in L-arginine which is the substrate for NO synthesis and its intake has been shown to improve FMD in healthy adults >70 years old [20]. Soy nuts also contain isoflavones (i.e., (poly)phenols), which are known to increase NO bioavailability and improve FMD [37,38]. These findings underscore the potential of certain (poly)phenol- and L-arginine–rich foods to enhance endothelial function in aging adults.

Foods that are rich in nitrates (e.g., beetroot) have been shown to benefit endothelial function by enhancing vasodilation and blood flow via the nitrate–nitrite–NO pathway [19,46,47]. In older adults with CVD risk factors, consuming beetroot gel two hours prior to testing significantly increased FMD compared to a placebo (7.09% vs. 4.00%) [22,40]. Similarly, a single dose of beetroot juice led to a significant FMD increase (Δ1.3% units) in older adults with peripheral artery disease, although individual responses varied, ranging from approximately 2% to 8% post-intervention [22,43]. In postmenopausal women with systemic arterial hypertension, one week of daily beetroot juice consumption significantly improved FMD compared to the control (7.35% vs. 2.85%) [44]. Benefits were also observed in early- and late-postmenopausal women, with beetroot juice increasing FMD by 0.53% and 3.72% units, respectively, from baseline [45]. Finally, consuming a high-nitrate salad twice daily for 10 days resulted in a significantly greater increase in FMD compared to the control (33% vs. 13% increase) in postmenopausal women [42]. These results highlight the potential of nitrate-rich foods as practical dietary strategies to enhance endothelial function in different aging populations.

Beyond individual foods, dietary patterns like the Mediterranean Diet (MED) and low-sodium diets have demonstrated benefits for endothelial function in aging adults [39,48]. An RCT comparing the MED to a habitual diet in healthy older adults showed a 1.3% unit increase in FMD after six months of the intervention [41,48]. The MED promotes a nutrient-dense diet that is rich in plant foods (e.g., containing (poly)phenols and nitrates), which may contribute to improved endothelial function through the mechanisms previously mentioned, as well as alternative pathways such as reducing inflammation. Similarly, a low-sodium diet (~1500 mg/day) in older adults with above-normal or stage 1 hypertension led to a 68% greater increase in NO-mediated FMD compared to those consuming a higher-sodium diet (~3600 mg/day) [39]. These results suggest that broader dietary patterns, such as the MED and low-sodium diets, can effectively support endothelial function in older adults.

## 4. Factors to Consider in Minimizing Flow-Mediated Response Variability

A key challenge for dietary interventional trials is the large variability in outcome responses among the participants, especially in aging adults. Response variability not only reflects the complex, multifactorial nature of aging itself but also encompasses other physiological, metabolic, and lifestyle factors that may be influencing individual responses. The following sections explore possible contributors to this variability in FMD responses and their implications for future research in precision nutrition for healthy aging.

### 4.1. Pre-Existing Endothelial Dysfunction

There are currently no standard reference values for FMD that are used to indicate endothelial dysfunction. However, researchers recently aimed to define FMD reference values and suggested that optimal endothelial function is an FMD ≥ 6.5% with two categories for dysfunction: impaired, which is from 6.4% to 3.8%, and pathological, which is ≤3.7% [49]. Compared to those who are considered to have optimal endothelial function, those with endothelial dysfunction might be more likely to respond to dietary interventions due to having a greater capacity for improvement. In a recent RCT, a mitochondrial targeted oral antioxidant (MitoQ, mitoquinone mesylate) improved FMD in middle-aged/older adults (≥45 years old) with a baseline FMD < 6% but not in adults with a baseline FMD > 6% [50]. Additionally, oral antioxidant (i.e., vitamin C, vitamin E, and alpha lipoic acid) supplementation significantly improved FMD acutely in older adults (71 ± 1 years) with endothelial dysfunction at baseline from 5.2% to 8.2%, while a decrease in FMD was seen in younger adults (25 ± 1 years) with normal endothelial function from 7.4% to 5.8% [12]. Future studies limited to individuals with impaired endothelial function (i.e., FMD < 6.5%) may provide better insight as to the degree of FMD responses to diet or supplementation interventions. Alternatively, assessing the change in FMD according to pre-specified reference values (i.e., pathological values of <3.8%, impaired values of <6.5%, optimal values of ≥6.5% [49]) may also provide insights for dietary intervention efficacy at varying levels of endothelial (dys)function.

### 4.2. Pre-Existing Comorbid Conditions

Pre-existing comorbid conditions may also explain dietary intervention response variability. In a systematic review and meta-analysis, (poly)phenol-based interventions improved FMD in only ‘at risk’ and diseased populations [51]. Specifically, a report from the CORDIOPREV study showed that the MED increased FMD after 1.5 years in patients with type 2 diabetes (3.8% to 5.2%) and prediabetes (3.8% to 4.9%), but FMD remained stable in patients without diabetes on the same diet [52]. While this study was not exclusively conducted utilizing older adults, the mean age was 59.5 ± 8.7 years, highlighting the possible effects in an aging population [52]. Dietary intervention trials assessing endothelial function improvement are typically conducted in populations with a specific clinical condition (e.g., overweight/obesity, hypertension, type 2 diabetes, low-grade inflammation), and/or they exclude participants with pre-existing conditions. Notably, 60% of adults >65 years old have at least one secondary pre-existing comorbid condition [53]. Recognizing and accounting for this in study design and/or analyses is critical to better explain FMD outcome response variability. For example, within specific disease states, co-occurring conditions, such as endothelial dysfunction, hypertension, and obesity, could be explored to understand how the broader comorbidity profile influences the variability in FMD responses to dietary interventions in older adults. Additionally, exploring post hoc analyses stratified on the degree and range of these different disease states relative to the outcome response might provide beneficial information for future precision nutrition approaches but of course requires large sample sizes that are often not feasible for randomized and controlled dietary intervention trials.

### 4.3. Menopause

Reproductive-aged women are more protected against endothelial dysfunction [54,55]. Estradiol levels stay constant until about the mid-40s, after which there is a sharp decline, reflecting diminishing follicle reserves. Estrogen is protective of the vascular system as it exerts antioxidant and anti-inflammatory properties and can bind to the estrogen receptors on endothelial cells promoting NO production [56,57]. Hence, as estrogen declines from pre- to postmenopause, there are observed reductions in FMD [45,56,58]. While beetroot juice improved FMD in postmenopausal women, FMD increased to a greater degree in late-postmenopausal women (>6 years following final menstrual period; 3.72% to 6.95%) than in early-postmenopausal women (1–6 years following final menstrual period; 5.13% to 5.66%) [45]. It is also important to note that both early- and late -postmenopausal women were classified as having endothelial dysfunction; however, late-postmenopausal women showed worsened FMD at baseline (5.13% vs. 3.72%) [45]. This may have been influenced by estrogen levels, which were not measured in the study, highlighting a potential direction for future research to better understand the role of estrogen in FMD responses to dietary interventions in postmenopausal women.

### 4.4. Dietary Adherence

It is crucial to understand whether adherence, or lack thereof, contributes to outcome variability in dietary intervention studies [59]. For instance, studies on beetroot and soy consumption in older adults mentioned above-quantified adherence through plasma nitrate/nitrite or serum isoflavone levels, respectively [36,60]. While such objective markers provide valuable insights, they only capture adherence at measurement time points and not throughout the study. Moreover, when adherence is averaged across participants from daily logs, studies may report the intervention group as “adherent,” without acknowledging that adherence levels are varying substantially among individuals. To gain a clearer understanding of how adherence influences response variability, future research should combine objective markers (e.g., metabolite concentrations) with subjective adherence tracking (e.g., daily diet logs). Additionally, subgroup analyses categorizing participants based on adherence levels, such as those with 100% adherence vs. partial adherence, could help clarify how adherence impacts FMD. It is possible that participants with full adherence (i.e., 100%) to a dietary intervention may experience significantly greater improvements in FMD compared to those with lower adherence, further emphasizing the need for more comprehensive adherence assessments in dietary intervention studies.

### 4.5. Microbiome

The microbiome plays a significant role in bioavailability and FMD response to dietary interventions, especially for the interventions that are high in nitrates and (poly)phenols. Nitrates begin being converted to NO in the mouth through the action of the oral bacteria, and the gut microbiome is the main source for (poly)phenol metabolism [61]. Thus, the baseline microbiome composition may impact or predict the outcome response, and the dietary intervention may change the composition of the microbiome [21]. For example, *Bifidobacterium* has been positively correlated with improved vascular function and reduced inflammation [62]. Wild blueberry supplementation significantly increased *Adlercreutzia*, *Bifidobacterium*, and *Dorea* amounts compared to the control in a diabetic mouse model [21]. Additionally, there may also be age-related alterations to the gut microbiome that should be considered [63]. Thus, analyzing the composition of the microbiome may be an essential component for understanding FMD response variability to dietary interventions that are especially high in nitrates and (poly)phenol compounds (e.g., fruits and vegetables).

### 4.6. Metabolomics

Metabolomics is a rapidly evolving field that provides valuable mechanistic insights into dietary intake and metabolism with the potential to explain interindividual variability in responses to dietary interventions and predict outcomes [64]. For example, baseline metabolites reflecting habitual diet have been shown to predict nitrite-induced increases in FMD following 10 weeks of oral nitrite supplementation in healthy middle-aged/older adults (50–79 years) [65]. Similarly, anthocyanin metabolites were correlated with FMD improvements following blueberry consumption in healthy older adults (65–80 years) [32]. It has been documented that individual metabolic profiles vary based on age, as well as other factors like health status and body mass index [64,66,67]. Thus, rather than simply reporting group-level averages of measured metabolites, understanding individual metabolic profiles or phenotypes, and how they relate to individual FMD responses, will deepen our understanding of possible mechanisms related to response variability. Additionally, combining metabolomic analyses with microbiome studies can provide further insights, as many bioavailable food compounds that are beneficial for FMD, particularly (poly)phenols, are heavily reliant on gut microbiota for metabolism.

### 4.7. Other Factors

Other factors that may contribute to variability in FMD responses to dietary interventions, particularly those that are relevant for the aging population, include physical activity, medication use, sleep quality, and hydration status. However, their specific impact on FMD variability in the context of dietary interventions in aging populations remains unclear, leaving a gap in our current understanding. Although these variables are often tightly controlled for or excluded in clinical trials, they are highly relevant in real-world aging populations. For example, physical activity decreases with age [68] and is a known modulator of endothelial function [69]. When endurance training was combined with estrogen supplementation, FMD increased to a greater extent than with estrogen supplementation alone in postmenopausal women [70]. Thus, physical activity may either complement or confound the vascular effects of dietary interventions in the aging population. Similarly, common medications in older adults (e.g., antihypertensives) have been shown to impact endothelial function [71] and/or interact with nutrients [72], thereby possibly influencing FMD responses to dietary interventions. Sleep disturbances and low-grade dehydration are also more prevalent with age [73,74] and have been associated with poorer FMD [75,76]. While all these additional factors may be tightly controlled through exclusion criteria, their potential influence on dietary intervention efficacy in aging populations should not be overlooked, and they may contribute to variability in FMD responses.

## 5. Conclusions and Future Directions

**Figure 2:**
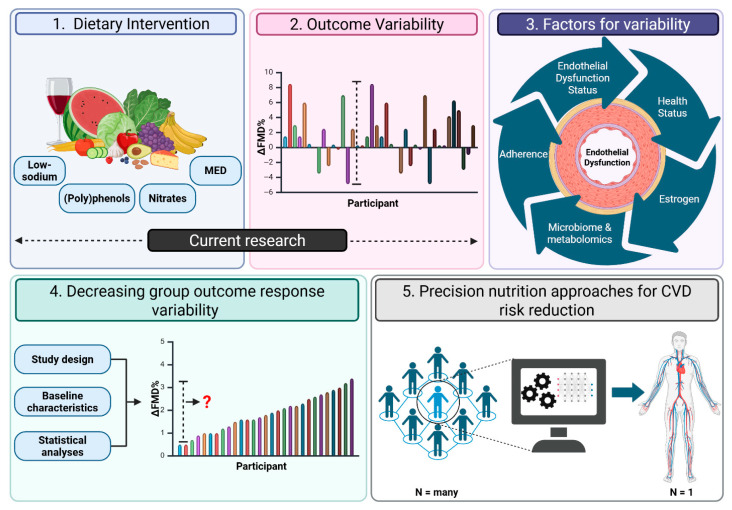
Conceptual framework for advancing precision nutrition approaches to improve endothelial function and reduce cardiovascular disease (CVD) risk in aging adults. Panels 1 and 2 illustrate current research demonstrating that while dietary interventions can improve flow-mediated dilation, substantial interindividual variability in responses remains a major challenge. Panels 3 and 4 highlight the integration of individual-level factors to explain and reduce this variability. Panel 5 represents the application of precision nutrition strategies guided by artificial intelligence and machine learning to tailor interventions and optimize vascular health outcomes. The data and visual elements in this figure are schematic and illustrative, not empirical. Abbreviations: flow-mediated dilation, FMD; cardiovascular disease, CVD.

Specific foods and dietary patterns have been shown to improve FMD in aging adults (Table 1). However, individual response variability remains a challenge in translating these findings into effective dietary guidance (Figure 2). While the present review provided studies demonstrating significant group-level improvements in FMD to establish that dietary interventions can be effective, countless studies in the broader literature report no group-level changes, potentially due to large individual variability that masks effects in some participants. Thus, rather than continuing to test different individual foods across varying doses or intervention durations, future research must prioritize identifying the specific individual-level factors that drive positive or negative FMD responses using dietary interventions that have already been shown to improve FMD in this population. Collective evidence highlighted in this review points to key contributors (e.g., baseline endothelial function, menopausal status, comorbidities) that may underline the variability in FMD outcomes among aging individuals (Figure 2).

To truly advance precision nutrition, future study designs should consider and integrate an array of diverse explanatory factors. However, comprehensively testing all these variables in a single RCT poses significant challenges. Narrowing participant pools by multiple phenotypic characteristics reduces generalizability and recruitment feasibility, while incorporating multi-omic analyses and adherence monitoring increases complexity, cost, and logistical demands. Therefore, it may not be realistic, or necessary, to include every factor in a single trial. Instead, a practical approach could involve carefully selecting and prioritizing key factors that are relevant to specific aging populations or interventions, employing stratified or adaptive trial designs, and leveraging advanced statistical methods (e.g., artificial intelligence and machine learning) to reduce variability and predict individual responses (Figure 2). Innovative studies, such as that conducted by Zeevi et al. (2015), show how rich individual-level data combined with machine learning and small-scale validation trials can guide the development of personalized dietary strategies [77]. Furthermore, sequential or multi-phase trials that combine mechanistic and clinical research can also build an evidence base for identifying predictive biomarkers and guiding personalized interventions.

By incrementally integrating comprehensive phenotyping into dietary intervention research, we can begin to reduce variability in outcome responses and move closer to achieving truly individualized strategies for improving vascular health in aging adults (Figure 2). While designing fully inclusive trials is daunting (e.g., multiple measurements, analyses, and a wide range of inclusion criteria), embracing this complexity is essential to identifying why aging individuals differ in their FMD responses to dietary interventions. This review focuses on reducing individual variability to strengthen the rigor of dietary intervention research which is foundational for the eventual clinical application of precision nutrition. A clearer understanding of these individual-level factors will not only improve study design and interpretation but also lay the groundwork for future translational efforts and more personalized approaches to cardiovascular prevention in clinical settings.

## Figures and Tables

**Table 1:** Randomized controlled trials (RCTs) that report dietary-induced improvements in endothelial function via flow-mediated dilation in aging adults.

Study	Study Design	Participants	Dietary Intervention	Control	Duration
Jablonski et al. (2013) Colorado, USA [39]	RCT, double-blind, crossover with 2-week washout	Middle-aged/older adults with high blood pressure (62 ± 7 y; BMI 27.1 ± 4.1 kg/m^2^) ^a^	Low sodium diet (1200 mg/day was the goal with anticipating actual mean intake of 1500 mg/day)	Normal sodium diet (3600 mg/day based on NHANES)	4 weeks
de Oliveira et al. (2016) Rio de Janeiro, Brazil [40]	RCT, double-blind, crossover with at least 1-week washout	Older adults with CVD risk factors (70.5 ± 5.6 y; BMI 30.2 ± 5.3 kg/m^2^) ^a^	100 g beetroot-based nutritional gel made from beetroot powder, and beetroot juice	100 g nitrate-depleted gel mixed with grated apple for similar texture, natural dye, and flavor to match beetroot-based gel	Single dose; measures 120 min post consumption
Davis et al. (2017) Adelaide, South Australia [41]	RCT, parallel arm	Healthy older adults (MED group: 71.0 ± 4.9 y and BMI 26.7 kg/m^2^; control group: 70.9 ± 4.9 y and BMI 27.1 ± 4.2 kg/m^2^) ^a^	MED (extra-virgin olive oil, F/V, whole grains, nuts, legumes, fish, and limited red meat and discretionary foods)	Habitual diet	6 months
Curtis et al. (2019) Norwich, UK [33]	RCT, double-blind, parallel arm	Older adults with overweight/obesity and metabolic syndrome (63 ± 7 y; BMI 31.2 ± 3.0 kg/m^2^) ^a^	26 g freeze-dried BB powder (364 mg ACN and 879 phenolics) added to foods	Placebo powder matched appearance and taste to the BB powder (0 mg ACN)	6 months
Mayra et al. (2019) Arizona, USA [42]	RCT, parallel arm	Postmenopausal women (52.6 ± 4.8 y; BMI 26.4 ± 6.4 kg/m^2^) ^a^	2 high-nitrate salads (celery, spinach, and romaine lettuce; 0.5 c or 15 g each; 284 mg nitrate)	1 cup (240 g) canned low-nitrate vegetables (green beans, corn, or green peas; ~30 mg nitrate)	10 days
Pekas et al. (2021) Nebraska, USA [43]	RCT, double-blind, crossover with 2-week washout	Patients with PAD (70.0 ± 7.0 y; BMI 29.1 ± 6.4 kg/m^2^) ^a^	Beetroot juice (0.11 mmol nitrate/kg)	Tapioca powder capsules (0 mg nitrate)	Single dose; measures 1 h post consumption
Tischmann et al. (2022) Maastricht, The Netherlands [36]	RCT, single-blind, crossover with 6–12 weeks of washout	Healthy older adults (64.1 ± 3.1 y; BMI 25.9 ± 2.8 kg/m^2^) ^a^	67 g unsalted soy nuts (~174 mg isoflavones)	No nuts	16 weeks
Wood et al. (2023) London, UK [32]	RCT, double-blind, parallel arm	Healthy older adults (BB group: 69.44 ± 3.48 y and BMI 24.57 ± 2.7 kg/m^2^; control group: 70.76 ± 3.81 y and BMI 23.16 ± 2.59 kg/m^2^) ^a^	26 g freeze-dried wild BB powder in water (302 mg ACN)	Isocaloric placebo powder matches the appearance and taste of BB powder (0 mg ACN)	12 weeks
Woolf et al. (2023) Colorado, USA [23]	RCT, double-blind, parallel arm	Postmenopausal women with high blood pressure (BB group: 60 ± 1 y and BMI of 27.6 ± 1.0 kg/m^2^; control group: 61 ± 1 y and BMI 27.8 ± 1.1 kg/m^2^) ^b^	22 g freeze-dried highbush BB powder in water (224 mg ACN, 726 mg total (poly)phenols)	Isocaloric placebo powder matches the appearance and taste to BB powder (0 mg ACN)	12 weeks
Benjamim et al. (2024) Ribeirao, Brazil [44]	RCT, triple-blind, crossover with 1-week washout	Postmenopausal women with systemic arterial hypertension (59 ± 4 y, BMI 29.2 kg/m^2^ ± 3.1 kg/m^2^) ^a^	70 mL beetroot juice (6.4 mmol or 400 mg nitrate)	70 mL nitrate-depleted beet root juice (0.38 mmol or ~40 mg nitrate)	6 days
Delgado Spicuzza et al. (2024) Pennsylvania, USA [45]	RCT, double-blind, crossover with 2-week washout	Early (1–6 y FMP) and late (>6 y FMP) postmenopausal women (56 ± 4 y and 63 ± 4 y, respectively) ^a^	140 mL beetroot juice (~9.7 mmol nitrate)	140 mL nitrate-depleted beetroot juice (~0.76 mmol nitrate)	Single dose; measures 100 min post consumption

Abbreviations: anthocyanins, ACN; blueberry, BB; body mass index, BMI; cardiovascular disease, CVD; final menstrual period, FMP; fruits and vegetables, F/V; Mediterranean diet, MED; National Health and Examination Survey, NHANES; peripheral artery disease, PAD; randomized controlled trial, RCT. ^a^ Data are represented as mean ± standard deviation. ^b^ Data are represented as mean ± standard error.

## Abbreviations

The following abbreviations are used in this manuscript:

CVD	Cardiovascular disease
FMD	Flow-mediated dilation
eNOS	Endothelial nitric oxide synthase
MED	Mediterranean Diet
NO	Nitric oxide
RCT	Randomized control trials
